# Frequent use of emergency departments and chronic conditions in ageing societies: a retrospective analysis based in Italy

**DOI:** 10.1186/s12963-020-00237-w

**Published:** 2020-11-09

**Authors:** Enrico di Bella, Luca Gandullia, Lucia Leporatti, Walter Locatelli, Marcello Montefiori, Luca Persico, Roberta Zanetti

**Affiliations:** 1 Department of Political Sciences, University of Genoa, Piazzale E. Brignole, 3A, 16124 Genoa, Italy; 2 Department of Economics and Business Studies, University of Genoa, Via Vivaldi 5, 16126 Genoa, Italy; 3 A.Li.Sa, Regione Liguria, Piazza della Vittoria, 15, 16121 Genoa, Italy

**Keywords:** Ageing, Emergency departments, Frequent use, Latent class model, Risk factors

## Abstract

**Background:**

Most western countries are facing relevant demographic changes, and the percentage of older people is destined to rise in the next decades. This fact is likely to affect the sustainability of healthcare systems significantly, mainly due to the connected issue of chronicity.

**Methods:**

In this paper, using an extensive and comprehensive administrative dataset, we analyse the phenomenon of frequent use of emergency departments (ED) in the oldest region in Europe (i.e. Liguria) over 4 years (2013–2016). Two alternative approaches are used to define categories of ED users based on the intensity and frequency of accesses and splitting patients into different age groups.

**Results:**

Results allow identifying clinical and socio-demographic risk-factors connected to different levels of ED utilisation and highlight the influential role played by chronic conditions (particularly mental disorders, respiratory diseases) and by multiple chronic conditions.

**Conclusions:**

The study aims at representing an informative tool to support policy-makers in setting proper policies addressed, on the one side, towards the potentially preventable frequent users and, on the other, towards those accessing due to complex medical conditions. The results can help in building a warning system to help general practitioners in the identification of potential frequent users and to develop preventive policies.

## Background

Emergency departments (EDs) are conceived to grant emergency care for acute and chronic illness and for various types of accidents and injuries. One of the main issues ED managers have to face in everyday activity is overcrowding, the situation in which the excessive number of patients requiring assistance exceeds the physical or staffing capacity of the ED or the hospital. In many cases, such an imbalance is due to ED accesses that are inappropriate or preventable through the definition of specific clinical paths or organisational changes [29, 37]. Numerous studies tried to define appropriate policy actions to contain this problem [10, 20, 28]. International literature identifies two main categories of ED users, whose accesses can be potentially avoided or reduced. The first group includes those who perceive EDs as a source of primary care alternative to general practitioner services. Those patients inappropriately use EDs for non-acute ordinary care that is out of the EDs’ true scope; patients accessing EDs inappropriately generally belong to similar socio-demographic groups and their frequent use is more often associated to foreign nationality, older age or disadvantaged economic and social condition [2, 8, 17]. The second group of EDs frequent users is composed of those who frequently access EDs due to a clinical condition that fosters their need for emergency services. Often, frequent users report a preponderance of chronic diseases, particularly asthma, renal failure, hypertension, and chronic pulmonary disease [4, 27]. Psychological distress [39] and alcohol and drug abuse [24] are also often associated with the frequent use of EDs. The number of patients suffering from chronic diseases is increasing due to the progressive ageing of the population in western countries and to the higher levels of urbanisation and pollution in eastern countries. This issue is likely to affect the demand for emergency services considerably and, more in general, for healthcare in the next decades. It is therefore not surprising to find that, although there is already a vast literature on how and why patients access EDs, there is still a strong focus on the subject. Despite this vast literature on the frequent use of ED and its determinants, there is still no consensus on how to define (theoretically and empirically) the concept of “frequent user” of EDs [14, 22]. Most of the previous studies define frequent or highly frequent users as those patients reporting several accesses per year beyond a threshold. The choice of which threshold values should be used to classify a patient as a frequent user is often subjective, and it is generally based on previous literature or some particular quantiles of the distribution of the number of ED accesses per patient in a given period [36, 43]. Three or 4 visits per year are the most commonly chosen thresholds, but various alternatives, from 2 to 12 visits per year, are also used [22]. Other approaches, which seek to avoid making the subjective choice of the threshold mentioned above, use finite mixture models to identify latent classes of users, including frequent users, based on medical care utilisation (e.g. [3, 12, 25]). This second category of works tries to identify “natural” clusters of patients according to how they access EDs.

A realistic scenario for ED usage is the coexistence of different types of patients: those who properly seek emergency care for unexpected acute health problems and those who, suffering from chronic diseases, will ask for assistance when the chronic diseases reach an acute phase. Among the patients that frequently access EDs, older people suffering from chronic diseases are a category of great interest. Ageing is one of the biggest challenges for western and advanced countries. In general, these countries are recording a steady reduction of mortality rates for all the age classes thanks to improvements in medical care and healthier lifestyles. For instance, the World Health Organisation Age Standardised Death Rate (ASDR) database^1^ shows that in Italy, the ASDR dropped from 700.4 death per 100,000 residents in 1980 to 351.9 in 2015. Analogous reductions in ASDR have been recorded for the main western/advanced countries such as the USA (ASDR 2016 = 480.2), UK (ASDR 2015 = 409.0), France (ASDR 2015 = 347.8), Germany (ASDR 2015 = 420.1) and Japan (ASDR 2015 = 302.0). The reduction in age-specific death rates, and consequently of ASDR, determines a larger share of people living longer. Reaching older ages determines a longer exposition to risk factors and the possibility of developing one or more chronic diseases. These are, by nature, long-term forms of disease that could be monitored and, if properly treated, should not determine frequent and inappropriate use of EDs.

With this paper, we aim at investigating the phenomenon of frequent use on the 2,512,025 accesses registered in the 19 EDs located in the Liguria Region (Italy) during the 2013–2016 period. From a demographic perspective, Liguria is a particularly interesting case study, because it is the oldest region in Europe in terms of percentage of people older than 65 (27% in 2017, compared to an EU average of 18%) and with 3.58 residents per 100,000 who live up to at least 105 years, against a national rate of 1.80. According to the Eurostat demographic projections, most of the EU contries will face a similar demographic structure in roughly 20 years. Consequently, analysing the Ligurian demand for emergency healthcare provides a clue on the possible evolution of healthcare demand in the next decades in most of the western countries.

This paper focuses on two possible strategies to identify patients that repeatedly access EDs, generating the negative effect of EDs’ inappropriate use and overcrowding. The first one is based on finite mixture models [12, 13] and the alternative one based on percentiles and the definition of thresholds. The first approach can be considered more effective for this goal, but it suffers from the significant limitation of being an ex-post approach. In other words, it requires the observation of the number of EDs accesses for each patient during the whole period of analysis. The second method is based on the simpler role of defining a threshold to classify patients according to the number of times they accessed to EDs. Even if this second approach suffers from the subjectivity in the threshold selection, it can be used “on the fly” as a warning system alerting general practitioners that their patients are accessing EDs with unusual frequency.

Also, compared to previous literature, the analysis benefits from the availability of data on 4 years and, consequently, it makes possible to define, not only frequent use but also the persistency of the frequent user status of patients over time. We differentiate between those patients who access several times but within a specific and delimited period (“one-shot” frequent users) and those who are “recurrent” or “persistent” frequent users [38]. Lastly, the analysis proposes a comparison based on different age groups: the behaviour of pediatric patients (0–14) is compared to that of adults (15+) and older patients (65+). Indeed, pediatric and older patients deserve specific attention because they show peculiarities in the characteristics of accesses. Even if a relationship between chronic diseases and the likelihood of repeated accessed also persists among children [1], the most relevant chronic conditions affecting children substantially differ from those affecting the adult population [30]. Besides, children’s ED utilisation is likely to be influenced by parents’ inexperience and apprehension or by their parents’ inability to assess pain in pre-verbal children [16, 35]

The prevention and control of chronic diseases is on the agenda of the world’s healthcare decision-makers [41]. Chronic pathologies represent the greatest burden in terms of health and economic costs. Indeed, chronic diseases are the leading causes of mortality and morbidity in Europe [7], and they absorb a relevant and increasing share of resources devoted to assistance, hospitalizations and drugs [6]. The continuous increase in the prevalence of cardiovascular diseases, diabetes and chronic respiratory diseases, in addition to the progressive ageing of the population in developed countries, calls for a revision of the healthcare model adopting a patient-centred approach. Tackling (often multiple) chronic diseases requires a reorganisation of primary care and the definition of effective strategies and monitoring tools in a long-term and integrated care model [42]. For all these reasons, the definition of a warning system as that proposed in this paper can become a precious tool to identify emerging critical conditions for specific patients. In fact, a relevant result of this paper in a health policy-making perspective is that the two statistical techniques considered bring to highly consistent results: the percentage of patients classified as frequent users by both the two methods is very high. This is an interesting result because, even though finite mixture models for frequent users identification are more precise from the statistical point of view, they can be hardly applied in real time.

On the contrary, the quantiles-based approach is much easier to implement, even in SQL-language based database systems, making it possible to implement an “on the fly” warning system. Such a system could alert general practitioners when some of the patients they assist access EDs in an “anomalous” way with two primary results on the health policy side. From one side, it may significantly reduce inappropriate accesses by patients that seek for primary and specialised care and, from the other, it can be used to detect a fast deterioration of chronic patients’ health conditions.

## Materials and methods

### Data

Statistical analyses are run on the administrative registry containing the records of 2,512,025 accesses^2^ made by 1,132,063 unique patients to the 19 EDs located in the Liguria Region (Italy) during the 4 years (2013–2016). The registry includes information on the demographic characteristics of each patient (year of birth, gender, nationality) and several fields connected to each access (e.g. time of arrival, time of first visit and dismissal, urgency level according to the triage system, clinical variables, means of arrival). The clinical variables are defined through the access diagnosis code that allows the identification of those patients suffering for specific chronic conditions. Diagnosis is classified according to the International Classification of Diseases, Clinical Modification (ICD-9-CM) coding system, which is an adaption of the International Statistical Classification of Diseases and Related Health Problems (ICD-9) introduced in the USA by the National Center for Health Statistics (NCHS). Five-digit ICD-9-CM diagnosis codes are used to classify accesses into acute or chronic ones. In detail, to identify 18 categories of chronic conditions, we used the approach of the Chronic Condition Indicator (CCI) developed as part of the Healthcare Cost and Utilization Project (HCUP), a Federal-State-Industry partnership sponsored by the Agency for Healthcare Research and Quality^3^. The CCI allows to categorize ICD-9-CM diagnosis codes into chronic (e.g. malignancies, diabetes, mental illness, hypertension, many forms of heart disease and congenital anomalies) or not chronic/acute conditions (e.g. infections, pregnancy, non-specific symptoms, injuries). In order to be defined as chronic, a condition should last at least 12 months and it should satisfy at least one of the following conditions: (1) causing limitations on personal-care, daily activities, and social life; and (2) resulting in the need for ongoing medical intervention [33]. The CCI indicator allows also to split chronic conditions according to the specific body systems affected in 18 different categories. All injuries are instead assumed to be connected to acute conditions.

Given these premises, each patient is defined chronic (using a dummy variable) if he/she has at least one access connected to a chronic diagnosis. Among the 18 categories of chronic conditions, we specifically focus on the most relevant ones in terms of the number of patients, that turn out to be he following: mental disorders, diseases of the nervous system and sense organs, diseases of the circulatory system, diseases of the respiratory system, diseases of the genitourinary system, diseases of the musculoskeletal system and congenital anomalies. Also, a dummy variable identifying those individuals suffering for more than one chronic condition (i.e. multi-chronicity) has been included in the analysis.

### A descriptive approach to model ED use

In the first step of our analysis, we classified patients in categories of users through the identification of a subjective threshold based on the number of accesses. Due to the wide time span of our records, it is evident that several accesses need to be associated with an intensity measure of the frequency of ED accesses during the period. For instance, eight accesses in 1 month are incomparable to eight accesses in 4 years. Similarly to the definition of frequent users given by Springer et al. [28], we split patients in those that disproportionally use ED just in a specific period, from those that disproportionally use emergency services over a more extended period. The 4 years have been therefore divided in semesters, differently specified for every single patient. For each patient, the first semester starts at the date of his/her first access; each access in the six months following the first access is considered as an access during the first semester; the second semester starts after 6 months from the starting date of the first access, and so on. At most, one patient can register eight semesters. According to this criterion, we define “frequent users” those patients who accessed any of the 19 ED considered more than three times in at least one semester. The aforementioned intensity measure is introduced considering the duration of the frequent user status: patients recording only one semester of frequent user status are classified as “one shot FU” whereas patients recording more than one semester of frequent user status are defined “multiple semester FU”.

### Modeling ED use through finite mixture models

The second step of our analysis involves the modeling of ED utilisation in a “data-driven” approach. From a theoretical point of view, access to medical care has been mainly investigated using two approaches. The first is based on the classical consumer theory [19] and considers the demand for healthcare services as determined by the consumers themselves. The second approach [44] relies instead on the principal-agent theory and claims that the consumption of medical care is determined by the supply (i.e. physicians influence patients’ demand). Under an econometric perspective, the two aforementioned theoretical approaches translate into the alternative use of finite mixture models (FMM) when Grossman’s model is preferred [12, 13] and hurdle models, when testing for principal-agent theory [18, 34]. In our context, access to EDs mainly falls under the Grossman’s approach since patients are free to choose “if and where” receive health services for deferrable cases. Clearly, in case of a real emergency, no other option than the nearest point is possible, and this is why we propose the adoption of the FMM approach to model count measures of health care use (e.g. the number of ED visits). Our dataset is limited to the population of ED users since the dataset does not include any information on the residents who recorded zero accesses during the study period. All the analyses are therefore designed to accommodate the presence of a zero-truncated dependent variable.

Several empirical applications support the use of finite mixture models in these contexts. The use of a finite mixture approach to model healthcare utilisation has a series of benefits over more traditional approaches (e.g. standard count models, two-part models) since it allows for additional heterogeneity among the populations of users. One of the first applications of finite mixture models in health economics has been proposed by Deb and Trivedi [12], who used such an approach to model the demand for medical care by elderly Americans. Results suggest that finite mixture model is more appropriate to model the unobserved heterogeneity (mainly determined by the latent health status) and support the idea that health care demand should be modelled appropriately under a consumer-theoretical framework rather than under a principal-agent setting (econometrically specified by the two-part approach). The methodology has been subsequently applied by several studies aimed at comparing the use of finite mixture and two-part models in the healthcare context, with a general favour for the former ones [11, 13, 21]. The consensus on the use of a finite mixture approaches in place of traditional ones also depends on its strong relation to latent class models. Indeed, finite mixture models allow to split patients into distinct classes of healthcare users (generally infrequent and frequent users), and this fact is generally preferred for its policy implications to the use of a two-part model that focuses on the distinction between users and non-users. Alternative approaches have been proposed to accommodate the panel structure of data [3] and for the zero-truncated dependent variables [25].

FMMs are used to classify observations, to adjust for clustering and to model unobserved heterogeneity. Observed data are assumed to belong to unobserved subpopulations (in our case “frequent user status” of patients), and mixtures of probability densities or regression models are used to model the outcome of interest. After fitting the model, class membership probabilities can also be predicted for each observation. The FMM proposed in this paper is a finite mixture approach that accommodates the presence of an integer and positive dependent variable representative of the ED utilisation (i.e. the number of accesses over the 4 years) and suspected unobserved heterogeneity [5]. Consider *y* to be the number of accesses that a patient registered of the period; they can be assumed to come from *K* distinct classes *f*_1_, *f*_2_, …, *f*_*k*_ in proportions *π*_1_, *π*_2_, …, *π*_*k*_. The finite mixture distribution is the weighted sum of component distributions:

$$ f(y)=\sum \limits_{h=1}^K{\boldsymbol{\pi}}_h\left(\boldsymbol{z},{\boldsymbol{\alpha}}_h\right){\boldsymbol{f}}_h\left(y;{\boldsymbol{x}}_h,{\boldsymbol{\theta}}_h\right) $$

where both the mixing probabilities ***π***_*h*_ and the component distributions ***f***_*h*_ may depend on two sets of relevant regressor variables **z** (with parameters ***α***_*h*_) and ***x***_*h*_ (with parameters ***θ***_*h*_). Component distributions ***f***_*h*_ might belong to different families.

Using the SAS FMM routine in SAS Studio, we estimated two sets of FMMs with three component distributions each^4^ for paediatric, adult and older patients: 3 Poisson distributions (as a base model) and 1 truncated Poisson distribution (to account for zero truncated values) and 2 Poisson distributions. In all the models, the determinants used to define latent classes are the ones given in Table 1 and belong to two domains: the socio-demographic and clinical characteristics of patients. The results discussed in the following are based on the best model, chosen according to the usual model selection metrics: AIC and BIC.

**Table 1:** Variables definition

Variable	Description	Domain
Male	Dummy equal to 1 if male	Socio-demographic
Foreign	Dummy equal to 1 if foreign	Socio-demographic
Age	Age at the time of the first access	Socio-demographic
Multichronicity	Dummy equal to 1 if suffering for multiple chronic conditions	Clinical
Mental disorders	Dummy equal to 1 if suffering chronic mental disorders	Clinical
Nervous system and sense organs	Dummy equal to 1 if suffering for chronic diseases of the nervous system and sense organs	Clinical
Circulatory system	Dummy equal to 1 if suffering for chronic diseases of the circulatory system	Clinical
Respiratory system	Dummy equal to 1 if suffering for chronic diseases of the respiratory system	Clinical
Genitourinary system	Dummy equal to 1 if suffering for chronic diseases of the genitourinary system	Clinical
Skin and subcutaneous tissue	Dummy equal to 1 if suffering for chronic diseases of the skin and subcutaneous tissue	Clinical
Musculoskeletal system	Dummy equal to 1 if suffering for chronic diseases of the musculoskeletal system	Clinical
Congenital anomalies	Dummy equal to 1 if suffering for chronic congenital anomalies	Clinical

## Results

### A descriptive classification of EDs users

Table 2 provides the distribution of patients per number of accesses over the 4 years. A very small number of patients are responsible for a larger share of accesses, and patients recording four or more accesses represent the 15.4% of patients but account for 42.3% of accesses. Moreover, the top 1% of patients (more than nine accesses) is responsible for approximately 9% of all the accesses.

**Table 2:** Distribution of accesses and patients over the period 2013–2016

Number of accesses	Patients	% Patients	Accesses	% Accesses
1	588,201	52.0%	588,201	23.4%
2	244,618	21.6%	489,236	19.5%
3	124,123	11.0%	372,369	14.8%
4	67,905	6.0%	271,620	10.8%
5	38,474	3.4%	192,370	7.7%
6	23,137	2.0%	138,822	5.5%
7	14,292	1.3%	100,044	4.0%
8	9,134	0.8%	73,072	2.9%
9	6,097	0.5%	54,873	2.2%
10	4,055	0.4%	40,550	1.6%
11	2,883	0.3%	31,713	1.3%
12	1,966	0.2%	23,592	0.9%
13	1,462	0.1%	19,006	0.8%
14	1,143	0.1%	16,002	0.6%
15	823	0.1%	12,345	0.5%
16	668	0.1%	10,688	0.4%
17	482	0.0%	8,194	0.3%
18	397	0.0%	7,146	0.3%
19	316	0.0%	6,004	0.2%
20+	1,887	0.2%	56,178	2.2%
Total	1,132,063	100%	2,512,025	100%

During the 4 years, 958,712 adult patients (15+) accessed the 19 Ligurian EDs, accounting for a total number of accesses equal to 2,129,074. Accesses connected to paediatric patients (0–14) were 382,951 referring to 173,351 patients.

**Fig. 1:**
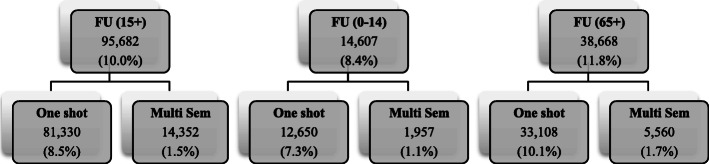
Definition of frequent users (FU) for adult (15+), paediatric (0–14) and older (65+) patients. The category of older patients (65+) is a subset of that of the adult patients (15+)

Figure 1 and Table 3 report a preliminary partition of patients according to the characteristics of EDs use. Frequent users are the 10% of the adult population (95,682 patients) and 8.4% (14,607) of children patients corresponding to slightly less than the 30% of adult accesses and roughly the 24% of paediatric accesses. Most of the frequent users are classified as one-shot FU (as defined above) while multiple semester FUs are less than 2% of patients regardless of age class (but among adult patients, they account for more than 9% of accesses).

**Table 3:** Characteristics of different categories of ED users

Variabile	Adult patients (15+)			Paediatric patients (0–14)			Older patients (65+)		
	Non FU	One Shot	Multi sem	Non FU	One Shot	Multi sem	Non FU	One Shot	Multi sem
% Patients	90.0	8.5	1.5	91.6	7.3	1.1	88.2	10.1	1.7
% Accesses	70.8	20.0	9.1	76.2	17.9	6.0	68.4	22.4	9.1
Average accesses	1.7	5.2	13.5	1.8	5.4	11.6	1.8	5.2	12.6
% Male	49.0	47.8	47.2	54.7	56.6	56.4	42.4	47.2	50.4
% Foreign	12.0	12.5	12.8	11.5	13.8	16.7	2.3	1.1	1.0
Average age	53.0	55.4	54.2	5.9	4.7	4.0	77.3	78.6	77.9
% Multiple chronic conditions	2.5	18.2	49.3	0.3	2.5	11.0	4.5	26.7	61.1
% Mental disorders	3.2	10.6	29.2	0.8	2.5	7.1	2.2	7.7	18.7
% Nervous system and sense organs	1.9	5.1	9.3	0.7	2.1	5.4	2.5	5.9	10.2
% Circulatory system	7.1	17.8	28.8	0.3	1.0	1.5	15.5	33.5	52.5
% Respiratory system	1.0	3.8	9.8	1.1	4.2	10.2	1.8	6.6	15.8
% Digestive system	0.7	2.3	6.0	0.2	0.8	1.6	0.9	2.7	6.1
% Genitourinary system	1.0	3.4	7.7	0.9	2.2	5.2	0.8	2.8	6.0
% Musculoskeletal system	1.2	3.2	7.0	0.5	1.5	2.6	1.6	3.8	8.5
% Congenital anomalies	10.3	25.6	46.1	4.0	9.2	16.6	11.7	28.6	14.1
Number of patients	958,712			173,351			328,901		
Number of accesses	2,129,074			382,951			765,971		

Looking at Table 3, it is immediately apparent that some socio-demographic and clinical characteristics are over-represented in the groups of frequent users of emergency services. If we consider adult patients, the percentage suffering for multiple chronic conditions account to 2.5% among occasional users (i.e. non-frequent users), but it rises to 49.3% among multiple semester frequent users; this percentage accounts to 61.6% if we consider older patients (65+). The preponderance of mental disorders, diseases of the circulatory system and congenital anomalies turn out to be particularly relevant in one shot and multiple semester frequent users. Within the paediatric population, diseases of the respiratory system are particularly relevant among frequent users, with a percentage of chronic patients among multi-semester frequent users equal to 10.2% compared to a 1.1% among occasional users. From a socio-demographic point of view, it is interesting to notice that foreign individuals are highly representative in the category of frequent paediatric users, while they represent a marginal share among older patients.

### Latent classes of users

The descriptive analyses in “A descriptive classification of EDs users” in the previous section identify the presence of a sharply uneven distribution of accesses with different patterns in the different age groups. However, they suffer from a subjective selection of the threshold in the determination of classes of users, possibly rising doubts about the actual reliability of the results. Consequently, in this section, we present the results obtained using a finite mixture approach that allows the definition of robust latent classes of users without determining the threshold a priori. Among the FFMs that have been estimated, the AIC and BIC values of the base (3 Poisson distributions) and chosen (1 truncated Poisson and 2 Poisson distributions) models are provided in Table 4 for the three age groups. The best models are the ones that include a truncated Poisson distribution among the components distributions.

**Table 4:** AIC and BIC values for a selection of finite mixture models

Component distributions	Adult patients (15+)	Paediatric patients (0–14)	Older patients (65+)
1 truncated Poisson and 2 Poisson	AIC: 2,711,550 BIC: 2,712,045	AIC: 513,881 BIC: 514,303	AIC: 958,152 BIC: 958,602
3 Poisson	AIC: 3,200,716 BIC: 3,201,294	AIC: 596,927 BIC: 597,349	AIC: 1,111,631 BIC: 1,112,081

The results of the FMMs with 1 truncated Poisson and 2 Poisson distributions on adult, pediatric and older patients are reported in Tables 5, 6 and 7, respectively. The estimated average value of the fitted number of accesses (reported in the last rows of the tables) helps in the understanding of the characteristics of ED’s use by patients belonging to the different latent classes. If we consider adults (Table 5), the first latent class identifies normal users (No FU) which comprise 84% of the patients and account for roughly the 69% of accesses (1.71 accesses per patient in average). Intermediate users represent instead 15% of patients and 28% of accesses, with an average number of accesses over the 4 years equal to more than 6. High users (i.e. those belonging to the third latent class) access to ED on average more than 18 times over the period and, even if they represent only the 0.5% of patients, they contribute to 3% of the accesses. Interestingly, among children, a lower percentage of patients belongs to the first class (78.8%), and the average number of accesses among high users belonging to the third latent class is significantly lower than that recorded for adults and older people (13.92). Older patients show an opposite situation in which also low users access almost two times over the period and middle users (i.e. second latent class users) represent 10% of patients but 27% of accesses.

**Table 5:** Parameter estimates for the three components truncated Poisson finite mixture model on adults (15+)

Variable	First latent class			Second latent class			Third latent class		
	Coef.	Std. err.	Sig.	Coef.	Std. err.	Sig.	Coef.	Std. err.	Sig.
Male	0.005	0.003		− 0.001	0.003		0.080	0.011	***
Foreign	− 0.137	0.006	***	0.022	0.005	***	0.035	0.015	*
Age	− 0.002	0.000	***	− 0.003	0.000	***	− 0.002	0.000	***
Mental disorders	0.736	0.005	***	0.650	0.006	***	0.758	0.015	***
Diseases of the nervous system and sense organs	0.542	0.006	***	0.357	0.009	***	0.245	0.021	***
Diseases of the circulatory system	0.616	0.005	***	0.413	0.006	***	0.295	0.016	***
Diseases of the respiratory system	0.634	0.007	***	0.468	0.010	***	0.248	0.023	***
Diseases of the digestive system	0.645	0.009	***	0.475	0.012	***	0.260	0.030	***
Diseases of the genitourinary system	0.640	0.008	***	0.435	0.010	***	0.392	0.023	***
Diseases of the musculoskeletal system	0.616	0.008	***	0.429	0.011	***	0.308	0.027	***
Congenital anomalies	0.693	0.004	***	0.495	0.005	***	0.418	0.012	***
Multiple chronicity	0.497	0.006	***	0.343	0.007	***	0.369	0.018	***
_cons	-0.166	0.006	***	1.432	0.006	***	2.446	0.017	***
Number of observations	958,712								
Correlation real accesses vs predicted	0.92								
AIC	2,711,550								
% of patients	84.4%			15.1%			0.5%		
% accesses	69%			28%			3%		
Average number of accesses	1.71			6.14			18.50		

Significance levels: *p value < 0.05; **p value < 0.01; ***p value < 0.001

**Table 6:** Parameter estimates for the three components truncated Poisson finite mixture model on paediatric patients (0–14)

Variable	First latent class			Second latent class			Third latent class		
	Coef.	Std. err.	Sig.	Coef.	Std. err.	Sig.	Coef.	Std. err.	Sig.
Male	0.101	0.009	***	0.040	0.007	***	0.045	0.021	*
Foreign	− 0.022	0.001	***	− 0.022	0.001	***	− 0.024	0.002	***
Age	− 0.113	0.015	***	0.068	0.010	***	0.059	0.029	*
Mental disorders	0.999	0.026	***	0.575	0.027	***	0.522	0.062	***
Diseases of the nervous system and sense organs	0.733	0.029	***	0.443	0.030	***	0.405	0.061	***
Diseases of the circulatory system	0.784	0.042	***	0.406	0.046	***	0.341	0.118	**
Diseases of the respiratory system	1.074	0.020	***	0.645	0.022	***	0.535	0.066	***
Diseases of the digestive system	0.781	0.047	***	0.381	0.057	***	0.217	0.115	
Diseases of the genitourinary system	0.863	0.025	***	0.441	0.029	***	0.389	0.066	***
Diseases of the musculoskeletal system	0.864	0.030	***	0.440	0.036	***	0.306	0.094	**
Congenital anomalies	0.809	0.015	***	0.455	0.014	***	0.507	0.038	***
Multiple chronicity	0.044	0.031		0.203	0.032	***	0.116	0.062	
_cons	− 0.110	0.011	***	1.4788	0.008	***	2.466	0.022	***
Number of observations	173,351								
Correlation real accesses vs predicted	0.95								
AIC	513,881								
% of patients	78.8%			20.4%			0.8%		
% accesses	61%			36%			4%		
Average number of accesses	1.58			5.41			13.92		

Significance levels: * *p* value < 0.05; **p value < 0.01; ***p value < 0.001

**Table 7:** Parameter estimates for the three component truncated Poisson finite mixture model on older patients (65+)

Variable	First latent class			Second latent class			Third latent class		
	Coef.	Std. err.	Sig.	Coef.	Std. err.	Sig.	Coef.	Std. err.	Sig.
Male	0.044	0.005	***	0.075	0.006	***	0.145	0.020	***
Foreign	− 0.772	0.030	***	− 0.252	0.021	***	0.216	0.072	**
Age	0.005	0.000	***	0.001	0.000	*	0.002	0.001	
Mental disorders	0.616	0.008	***	0.555	0.012	***	0.672	0.033	***
Diseases of the nervous system and sense organs	0.458	0.009	***	0.312	0.015	***	0.251	0.045	***
Diseases of the circulatory system	0.591	0.006	***	0.400	0.007	***	0.282	0.027	***
Diseases of the respiratory system	0.562	0.009	***	0.440	0.013	***	0.261	0.044	***
Diseases of the digestive system	0.601	0.013	***	0.481	0.020	***	0.319	0.091	**
Diseases of the genitourinary system	0.467	0.013	***	0.297	0.019	***	0.140	0.058	*
Diseases of the musculoskeletal system	0.547	0.011	***	0.415	0.015	***	0.416	0.046	***
Congenital anomalies	0.631	0.006	***	0.482	0.008	***	0.514	0.025	***
Multiple chronicity	0.521	0.009	***	0.317	0.011	***	0.269	0.036	***
_cons	− 0.557	0.023	***	1.180	0.031	***	2.124	0.113	***
Number of observations	328,901								
Correlation real accesses vs predicted	0.91								
AIC	958,152								
% of patients	89.6%			10.1%			0.3%		
% accesses	71%			27%			2%		
Average number of accesses	1.84			6.27			18.32		

Significance levels: *p value < 0.05; **p value < 0.01; ***p value < 0.001

A first look at the tables shows that most of the variables have the expected sign and are statistically significant. This is not surprising also given the large datasets that we are analysing. However, the effects of the covariates are different across the different latent classes. Among adults, the socio-demographic determinants have a differentiated impact in the different latent classes: male frequent users tend to access ED more often than females while gender is not relevant for the other categories of users. Foreign frequent and highly frequent users tend to use emergency services more often than native-born patients, while age affects negatively and significantly the accesses in all the classes of users. From a clinical point of view, individuals reporting chronic conditions access to EDs more often, and the impact of suffering for mental disorders is the strongest among highly frequent users (i.e. third latent class). On the contrary, suffering from chronic diseases of the musculoskeletal system positively and strongly affects the number of accesses among low users. Finally, suffering from multi-chronic conditions impacts positively on the frequency of access of all users.

Looking at the paediatric population (Table 6), among socio-demographic drivers, age has negative impact among all users (i.e. higher age individuals record lower number of accesses), and this can be a consequence of the more frequent use of EDs during the first months of life. Some chronic conditions turn out to be particularly relevant in affecting the number of accesses, particularly diseases of the digestive system among low and frequent users and diseases of the respiratory system, think for example to paediatric Asthma [15], diseases of the musculoskeletal system, diseases of the digestive system and congenital anomalies. As expected, the presence of multiple chronic conditions has a much lower (often not significant) impact since it represents a rare condition when focusing on children.

Turning to the last category of patients (older than 65) (Table 7), we notice that age has an opposite impact compared to that found out for adults and children. Indeed, being older impacts positively to the number of accesses of all latent classes, even if not significantly among frequent users. The impact of being foreign is also controversial since it impacts negatively on accesses among patients belonging to first and second latent classes; this can be a consequence of the relatively low presence of older foreign people in the region. The coefficient related to multiple chronic conditions is positive and significant, suggesting that their number of accesses is significantly higher in the presence of comorbidities. The chronic conditions more influential in determining an increase in accesses are mental disorders, diseases of the circulatory system, congenital anomalies.

The estimation of predicted posterior probabilities of class membership for each patient^5^ allows us to detect further the intrinsic characteristics of each latent class (Table 8). For the sake of brevity, Table 8 reports a selection of some variables of interest. Interestingly, among older patients, 19% of those belonging to the third class suffer for more than one chronic condition, whereas the same percentage is equal to 7% in the first latent class and 10% in the second. A similar pattern is recorded for adults, whereas among children, as expected, the presence of multiple chronic conditions is not relevant. Another characteristic that seems to be over-represented among higher users is the foreign nationality, particularly among adults: 14.7 % of patients in the third latent class are foreign compared to 11.9% in the first class. Lastly, we investigate the urgency of accesses: in particular compute, for each patient, the percentage of non-urgent accesses over the total number of accesses. We consider non-urgent accesses those classified as white or green colour in the triage system. Interestingly, a significantly higher percentage of patients classified as high users tend to use emergency services for non-urgent (i.e. not life-threatening) conditions. This suggests that inappropriate use may be an issue among highly frequent users.

**Table 8:** Posterior estimations of the prevalence of specific characteristics

Variabile	Adult patients (15+)			Paediatric patients (0–14)			Older patients (65+)		
	1st latent class	2nd latent class	3rd latent class	1st latent class	2nd latent class	3rd latent class	1st latent class	2nd latent class	3rd latent class
% Multiple chronicity	4.2	7.0	13.8	0.5	1.1	2.5	7.3	10.2	18.9
% Foreign	11.9	13.0	14.7	11.4	13.4	13.6	2.3	2.0	2.2
% Non-urgent accesses	72.4	73.9	75.8	90.7	91.2	91.5	56.5	58.6	67.7

### Different definitions of frequent users: a comparison

In this section, we provide a comparison of the results obtained under the two approaches. In particular, we aim at comparing the classification of users defined in terms of latent classes with that obtained from the definition of the subjective threshold. Table 9 shows (for the three age groups and each latent class) how patients are distributed according to the first definition adopted (Non-FU, One-shot FU, Multiple semester FU). It is immediately evident that the two classifications are fairly consistent: if we focus on adults, the 95% of patients belonging to the first latent class are classified as non-FU, while slightly less than 80% of those belonging to the third latent class are multiple semester frequent users. The same pattern is verified for children and older patients. The less consistent classification is that of patients belonging to the second latent class (“intermediate users”) that tend to be split among non-frequent users and one-shot frequent uses.

**Table 9:** Matching between the two classifications (i.e. latent classes and frequent users categories) measured as the percentage of patients belonging to each latent classes located in the different frequent users’ categories

		Non-FU	One-shot FU	Multiple semester FU	Total
15+	First latent class	95.31	4.39	0.29	100
	Second latent class	46.63	43.90	9.47	100
	Third latent class	1.05	19.88	79.06	100
0-14	First latent class	97.65	2.31	0.04	100
	Second latent class	59.72	35.56	4.72	100
	Third latent class	2.85	25.97	71.18	100
65+	First latent class	93.51	6.04	0.44	100
	Second latent class	43.66	45.54	10.81	100
	Third latent class	0.73	18.09	81.17	100

## Discussion

The progressive ageing of the population in most western societies and the strict government’s budget constraints are leading to an increasing concern about the future sustainability of healthcare systems, especially for what concerns National Healthcare systems like the Italian one. Ageing and chronicity are indeed two sides of the same coin and, in all ageing countries, we may expect an explosion of healthcare resources absorbed by chronic patients during the next decades. This issue does not only involve the oldest region in Itlay (i.e. Liguria) but it should cause concern in most OECD countries. On average across 27 OECD countries [32] almost one third of people over 15 years old suffers from more than one chronic conditions and this percentage is destined to rise as a consequence of ageing. Today, on average, the percentage of older individuals (65+) living with more than one chronic conditions account to 58%, reaching more than 70% in several OECD countries. The analysis of the demand for healthcare services is indeed crucial to identify needs and challenges for the next decades around OECD countries. The approach proposed in this paper can be easily extended to all the countries characterized by ongoing deep demographic changes, also thanks to the increasing availability of large administrative data for research purposes.

Frequent use of EDs causes two main problems; the first is connected to extra costs for ED activity, the second is the risk to compromise the quality of the services provided because of overcrowding. Concerning ED activity, the primary determinant of quality is probably the speed at which the patient is taken in charge. Excessive flow of patients may negatively affect the timeliness of the treatment and of hospital admission, with particular reference to high severity patients for which a delay in the treatment they require could drive to dramatic consequences. Furthermore, overcrowding creates stress in ED activity that could, in turn, boosts the risk of errors.

Our investigation, by a positive approach, highlights the main determinants of frequent use and identifies those categories of people more likely to be frequent users. For instance, it is interesting to notice that, from a quantitative point of view, the category of “intermediate users” is very relevant in terms of impact on the total number of accesses: they contribute to roughly 30% of accesses among adults. Consequently, a more in-depth analysis of people belonging to this category can lead to a significant reduction in their number of accesses with a connected reduction in waiting times and overcrowding. Switching from a positive to a normative perspective, our study makes at the policy-maker disposal useful and informative tools to implement effective policies suitable for better management of patient flow.

By our empirical analysis, the policy-maker could target specific policies aiming at reducing the inappropriate use of EDs. One aspect that is pointed out by the analysis is the fact that foreign adult individuals tend to use more often EDs. This is probably due to the poor knowledge of the alternative services available for primary care (e.g. general practitioner) or to objective difficulties in accessing to other local health services (to which sometimes they have no right to access because of their condition, such as, for instance, their residence permit). This issue may be mitigated through informative campaigns addressed to them or by available territorial health services to which they can be admitted. In general, according to Cho et al. [9] improving health literacy may lead to progress in the health status and, consequently, to a reduction in hospital and emergency room use also among elderly patients. Another point that is highlighted by our investigation is the crucial role played by chronic conditions in affecting ED use: despite the medical need that brings chronic patients to visit EDs, the suggested analysis can help in the identification of clinical pathways (specific for specific pathologies) that can help patients to reduce their need for emergency services by increasing the effectiveness of treatments. Indeed, an inappropriate or incomplete treatment of chronic patients may lead to a fast deterioration of patients’ clinical condition, boosting their need for emergency services. It is highly recognized that the treatment of chronic patients should pass through a holistic approach that puts at the centre the single patient [42]: compliance to therapy, prevention and healty lifestyles are indeed crucial allies in the fight against several chronic conditions (e.g. diabetes, cardiovascular diseases). Therefore, general practioners can become foundamental vehicle of health, especially for older and multi-chronic patients. The strengthening of community care and primary care services (e.g. extending primary care opening hours) can help in the reduction of the use of EDs and hospitals [23, 26, 40]. Across EU, admissions connected to diabetes, hypertension, heart failure chronic obstructive pulmonary disease/bronchiectasis and asthma absorbed 37 million bed days in 2015, and most of them are estimated to be potentially avoidable through better prevention and disease management [31].

Coming back to our findings, from a policy perspective, general practitioners can become precious agents in reducing excessive use of EDs. Indeed, more than 200,000 accesses are attributable to multiple semesters frequent users; more than 139,000 of these accesses are recorded after the first semester of frequent use. This means that proper warning systems can be implemented to help general practitioners in the identification of potential multiple semesters frequent users to adopt suitable policies aimed at preventing their future ED use. Such a warning system may suggest that general practitioners contact their patients to understand why they are over-accessing EDs and act accordingly. The proposal of “meeting people’s needs outside of the hospital” [31] has been already accepted by some coutries developing specific services (e.g. call centres or home care services) for people recently discharged or at high risk of hospital (re-)admission [31]. Developing a tool that allows preventing the frequent use of EDs may reduce overcrowding, admissions and healthcare costs and improve the patients’ health status.

## Conclusions

The primary purpose of this paper was to model the utilisation of EDs in the oldest region in Europe (Liguria) to detect its most relevant determinants among clinical and socio-demographic drivers and to have new clues on the evolution of medical emergency needs around Europe. The analysis, carried on by using administrative data over 4 years, clearly shows the role played by frequent users: a small number of people determines a vast number of accesses to EDs. The finite mixture approach used allows classifying patients in three latent classes of users for different age groups (15+; 0–14; 65+) identifying the impact of different risk factors. The two approaches used in the identification of classes of users turn out to be very consistent and this suggest that most of the observations are correctly classified also following the descriptive approach. Both the finite mixture model and the descriptive analysis allow to identify potential risk factors predictive of disproportional use of ED; this information tool can be used by policy-makers to anticipate the needs of specific categories of patients and to prevent them from accessing EDs using instead alternative healthcare services.

## Data Availability

The datasets that support the findings of this study are available from Regione Liguria, but restrictions apply to the availability of these data, which were used under license for the current study, and so are not publicly available.

## Abbreviations

AIC: Akaike’s Information Criterion
BIC: Bayesian Information Criterion
CCI: Chronic condition indicator
ED: Emergency department
FU: Frequent user
FMM: Finite mixture model
NCHS: National Center for Health Statistics

## Footnotes

1 https://apps.who.int/healthinfo/statistics/mortality/whodpms/

2 . The original dataset contains 2,519,511 observations. However, some cleaning procedures have been developed to identify possible recording mistakes (e.g. patients recording different year of birth, patients with missing information) and to cut for outliers (i.e. patients having several accesses higher than 100 over the period). This last category of patients is indeed likely to be ascribable to the use of “fake” patients’ identifier number used, for instance, for recording not-registered foreign patients.

3 https://www.hcup-us.ahrq.gov/toolssoftware/chronic/chronic.jsp

4 More alternative models have also been considered: four components FMMs degenerated in three components FMMs whereas other assortments of distributions (e.g. negative binomial distribution to account for overdispersion) did not bring the model to convergence or did not provide any improvement in the model goodness of fit.

5 The posterior probability of class membership has been obtained by classifying each observation in the latent class in which the highest posterior probability is recorded. The posterior probabilities are a function of the latent-class predictors and the fitted outcome densities.
